# Relationships of Insulin Action to Age, Gender, Body Mass Index, and Waist Circumference Present Diversely in Different Glycemic Statuses among Chinese Population

**DOI:** 10.1155/2018/1682959

**Published:** 2018-08-23

**Authors:** Shao-jie Pang, Qing-Qing Man, Shuang Song, Peng-Kun Song, Zhen Liu, Yu-Qian Li, Shan-Shan Jia, Jing-Zhong Wang, Wen-Hua Zhao, Jian Zhang

**Affiliations:** National Institute for Nutrition and Health, Chinese Center for Disease Control and Prevention, 27 Nanwei Road, Xicheng District, Beijing 100050, China

## Abstract

**Introduction:**

To study the influence of different glycemic statuses on the relationship of insulin action to age, gender, body mass index (BMI), waist circumference (WC), and waist-to-height ratio (WHtR) among Chinese population.

**Methods:**

A total of 35,327 participants (17,456 males and 17,871 females) were included in this nationally representative cross-sectional study. Glycemic status was defined according to the 2010 American Diabetes Association criteria. Fasting insulin was measured by the chemiluminescence method.

**Results:**

Insulin and HOMA-IR levels were the highest in newly diagnosed diabetes and were lowest in normal fasting glucose (NFG) (*P* < 0.001). Insulin and HOMA-IR levels were higher in females (*P* < 0.001) than in males with previously diagnosed diabetes and impaired fasting glucose (IFG) and NFG, meanwhile decreased with age (*P* < 0.001) among IFG and NFG participants. As compared with participants with a BMI from 18.5 to 19.9, those in the lowest BMI category (<18.5) had a significantly elevated risk of IR (OR, 1.96; 95% CI, 1.01–3.80), as did those in the higher BMI categories among NFG participants. The risk of IR increased with WC and WHtR, and the response was linear (*P* < 0.001 for linear trend) for the participants with NFG but not in those with IFG.

**Conclusions:**

Different glycemic statuses significantly affect the relationships of insulin action to age, gender, BMI, WC, and WHtR among Chinese population.

## 1. Introduction

The prevalence of diabetes has risen dramatically over the past thirty years in China, which reached 10.9% in 2013 among Chinese adults [1, 2]. The increased diabetes epidemic is closely linked to the upsurge in adiposity [3, 4]. Adiposity causes sustained increase in serum free fatty acid which results in the systemic insulin resistance (IR) [5]. Synergy of adiposity and IR may further increase the risk for type 2 diabetes mellitus (T2DM) [6].

The homeostasis model assessment for IR (HOMA-IR) index has been widely used as a measure of IR in clinical practice and large epidemiological studies [7, 8]. Determining the insulin level and HOMA-IR in different glycemic statuses is vital to help clinicians interpret their value. At the same time, significant differences among subgroups stratified by gender and age should be considered [9]. Some studies have reported that serum insulin levels showed higher in females and HOMA-IR levels decreased with age, whereas others reported that insulin levels were higher in males and the relationship between insulin action and age was not statistically significant [10–14]. To our knowledge, these studies did not consider the different glycemic statuses. One study has shown that the levels of serum insulin and HOMA-IR were distinguishing in different glycemic statuses [15].

T2DM is a disease closely related to lifestyle, and adiposity is a key factor in the development of T2DM [16, 17]. It is estimated that about 90% of T2DM is attributable to overweight [18]. Body mass index (BMI), waist circumference (WC), and waist-to-height ratio (WHtR) have been proposed to assess adiposity and as independent predictors of diabetes [19, 20]. Some studies have suggested that HOMA-IR levels increased significantly with the increase of BMI and body fat percentage (BF%), and IR was strongly related to high BMI and high BF% [21, 22].

Up to now, there is no large epidemiological study to investigate the relationship of insulin action to age, gender, BMI, WC, and WHtR in different glycemic statuses in Chinese population although the prevalence of diabetes increased sharply. Therefore, our study aims at exploring the influence of different glycemic statuses on the relationship of insulin action to the related factors in Chinese population based on the data of nationally representative cross-sectional survey in 2015.

## 2. Study Participants, Materials, and Methods

### 2.1. Study Participants

The nationwide cross-sectional study of China National Chronic Diseases and Nutrition Survey (2015) was conducted by Chinese Center for Disease Control and Prevention to assess the nutrition and health status of Chinese population. Based on the national mortality surveillance system that included 605 surveillance points covering 24% Chinese population and major geographic regions of all 31 provinces, autonomous regions, and municipalities directly under the central government throughout China, this survey selected 302 surveillance points. The first level of sampling was stratified by 31 provinces. Within each province, 8 strata were then generated according to the proportion of urban population (high/low), overall population size (high/low), and mortality rate (high/low). The second level of sampling was stratified by urban and rural locations.

At each surveillance site, participants were selected by a complex and multistage probability sampling design. Firstly, we selected 3 urban subdistricts or rural townships from each surveillance site with probability proportional to size. Secondly, we selected 2 neighborhood communities or administrative villages with probability proportional to size. Then, 45 households were randomly selected from neighboring communities or administrative villages. Survey questionnaires, physical examination, and fasting blood collection were conducted for all the participants aging 18 years old and above. A total of 35,327 participants (17,456 males and 17,871 females) were included in this study.

Data were collected by trained staff in health examination centers from local health stations or community clinics according to a standard protocol. Questionnaires handed out by trained interviewers referred to demographic information, medical history, and lifestyle factors. Current smoking referred to “having smoked 100 cigarettes during lifetime” and “currently smoking.” Current drinking was referred to “alcohol intake more than once per month during the past 12 months.” Height was measured with no shoes to the nearest 0.1 cm. Weight was measured without shoes but in light clothes to the nearest 0.1 kg. Body mass index (BMI, kg/m^2^) was calculated as weight (kg) divided by the square of height (m). Waist circumference (WC) was measured on midway between the lower edge of the costal arch and the upper edge of the iliac crest among standing participants. Waist-to-height ratio (WHtR) was calculated as the ratio of WC to height. Blood pressure (mmHg) was measured at the nondominant arm with seated participants after 5 minutes of rest, for 3 times in succession with 1-minute interval between the measurements. We used an automated device for this measurement (OMRON Model HEM-7071, Omron Co.).

The study protocol was approved by the ethical review committee of the Chinese Center for Disease Control and Prevention. Written informed consent was obtained from all participants.

### 2.2. Clinical and Biochemical Methods

This study collected blood samples from all participants undergoing an overnight fast of 10–14 hours. Samples were centrifuged at 1500 rpm for 10 minutes after being left standing for 30 to 60 minutes. The centrifuged serum samples were then transported to the center laboratory of the Chinese Center for Disease Control and Prevention and stored at −80°C. Fasting blood glucose (FPG), total cholesterol (TC), low-density lipoprotein cholesterol (LDL-C), high-density lipoprotein cholesterol (HDL-C), and triglycerides (TG) were measured by the Hitachi 7600 automatic biochemical analyzer (Hitachi Inc., Tokyo, Japan) with reagents from Wako Pure Chemical Industries Ltd. (Tokyo, Japan) [23]. HbA1c was assessed by high-performance liquid chromatography (Premier Hb9210). Chemiluminescence method was adopted to measure fasting insulin (Roche E601). The insulin resistance index (homeostasis model assessment of insulin resistance, HOMA-IR) was calculated as fasting insulin (*μ*U/ml)^∗^FPG (mmol/L)/22.5.

### 2.3. Definition of Glucose and Insulin Resistance Status

Previously diagnosed diabetes referred to “diabetes (T2DM) had been diagnosed by physician previously.” According to the ADA 2010 criteria [24], newly diagnosed diabetes referred to FPG level ≥ 7.0 mmol/L and/or HbA1c concentration ≥ 6.5%. Impaired fasting glucose (IFG) was defined as FPG levels of 5.6~6.9 mmol/L and/or HbA1c concentrations of 5.7%~6.4%. Normal fasting glucose (NFG) was defined as FPG levels < 5.6 mmol/L and HbA1c concentrations < 5.7%. IR was defined using HOMA-IR > 95th percentile for normal fasting glucose (NFG) and HOMA-IR > 90th percentile for IFG.

### 2.4. Statistical Analysis

Data analyses were conducted adopting SAS version 9.4 (SAS Institute Inc., Cary, NC, USA). All values were presented as mean or percentage (95% confidence interval, 95% CI). Due to skewed distribution, logarithmically transformed values of fasting insulin and HOMA-IR were adopted. Differences in continuous variables of glycemic status were assessed by one-way analysis of variance (ANOVA) with subsequent Bonferroni correction for multiple comparisons. Differences in proportions between the glycemic status groups were examined by *χ* ^2^ analysis. Differences in the level of serum insulin and HOMA-IR between the genders were measured with analysis of covariance (ANCOVA) adjusted for age, FPG, and HbA1c.

Multiple linear regression was adopted to explore the independent effects of age for serum insulin and HOMA-IR levels. Three models were model 1 adjusted for education (less than high school, high school or equivalent, or college or above), marital status (married or unmarried including single, widowed, or separated), smoking status (current smoker, former smoker, and never smoked), alcohol consumption (currently, formerly, and never), physical activity (yes or no), TC, TG, LDL, and HDL; model 2 adjusted for all variables in model 1 plus diabetes history in family (yes or no), glucose, and HbA1c; and model 3 adjusted for all variables in model 2 plus BMI and WC.

Multivariable-adjusted logistic regression was performed to explore the effects of BMI, WC, and WHtR on HOMA-IR. BMI was classified as <18.5, 18.5–19.9, 20.0–21.9, 22.0–23.9, 24.0–25.9, 26.0–27.9, 28.0–29.9, and ≥30. WC was classified as <70, 70–74, 75–79, 80–84, 85–89, 90–94, 95–99, and ≥100 for male (<65, 65–69, 70–74, 75–79, 80–84, 85–89, 90–95, and >95 for female). WHtR was classified as <0.41, 0.41–0.43, 0.44–0.46, 0.47–0.49, 0.50–0.52, 0.53–0.55, 0.56–0.58, and ≥0.59. Odds ratios (OR) and 95% CI were measured. The group of the lowest ratio of insulin resistance was set as the reference. *P* < 0.05 was set statistically significant.

## 3. Results

Table 1 shows the demographic and clinical characteristics of the participants in this study. The mean age of 35,327 participants in total was 56.2 years. Of these, 4360 (46.9% males) were previously diagnosed T2DM, 4433 (51.1% males) were newly diagnosed T2DM, 7438 (49.4% males) participants with IFG, and 19,096 (49.6% males) with NFG. Mean age was the highest in previously diagnosed diabetes participants compared to the other groups while HDL was the lowest (*P* < 0.001). DBP, TC, TG, LDL, and FPG were highest in those with newly diagnosed diabetes than the other groups (*P* < 0.001). The insulin and HOMA-IR levels were the highest in newly diagnosed diabetes participants and were lowest in NFG (*P* < 0.001). Insulin levels were higher in IFG than in previously diagnosed diabetes participants (*P* < 0.001), whereas HOMA-IR levels were the opposite (*P* < 0.001).

**Figure 1:**
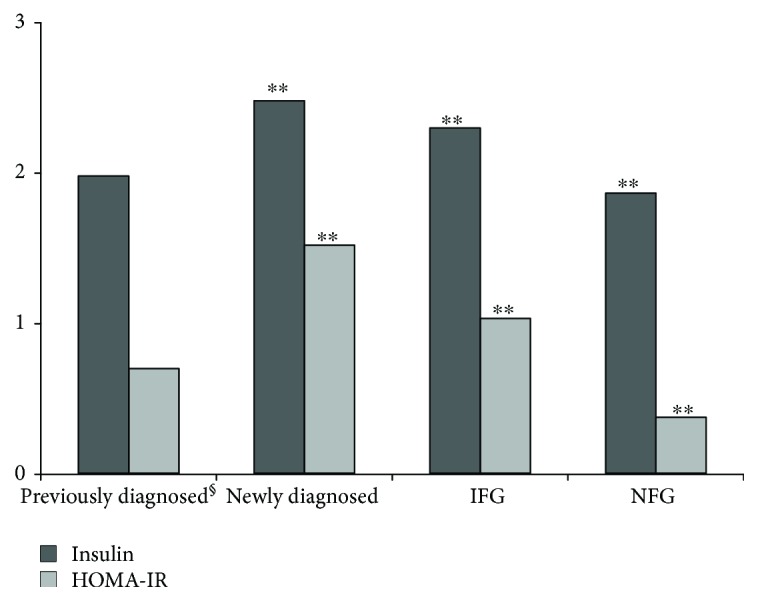
Level of insulin and HOMA-IR by glycemic status. ^§^ *N* = 407, excluding the participants who control blood glucose (including oral hypoglycemic drug, insulin injection, diet control, and exercise); compared with previously diagnosed, ^∗∗^ *P* < 0.001.

When participants who controlled blood glucose in previously diagnosed diabetes were excluded, the concentration of FPG was 9.7 mmol/L and HbA1c was 7.1%, both of them were higher than in other groups (*P* < 0.001). Moreover, the insulin level and HOMA-IR in the participants with previously diagnosed diabetes were higher than in those with NFG but lower than in the groups of newly diagnosed diabetes and IFG (*P* < 0.001, Figure 1).

Among participants with previously diagnosed diabetes, IFG, and NFG, insulin and HOMA-IR levels were higher in females than in males (*P* < 0.001, Table 2). For newly diagnosed diabetes participants, HOMA-IR levels were higher in females than in males (*P* = 0.012). However, there was no difference for insulin levels between the genders (*P* = 0.101).

Insulin and HOMA-IR levels decreased with age (*P* < 0.001) in both males and females for IFG and NFG participants (Table 2). In newly diagnosed males, levels of insulin and HOMA-IR were negatively related to age (*P* < 0.001). In newly diagnosed females, the correlation of insulin levels with age was no statistical significance after multivariate adjustment (*P* = 0.118). However, the inverse relationship between HOMA-IR levels and age was observed (*P* = 0.023). For previously diagnosed diabetes participants, the relationship of insulin and HOMA-IR levels with age was not significant after multivariate adjustment. In the sensitivity analysis, it was still not significant for the relationships of insulin and HOMA-IR levels with age when excluding insulin injection (Table S1).

**Figure 2:**
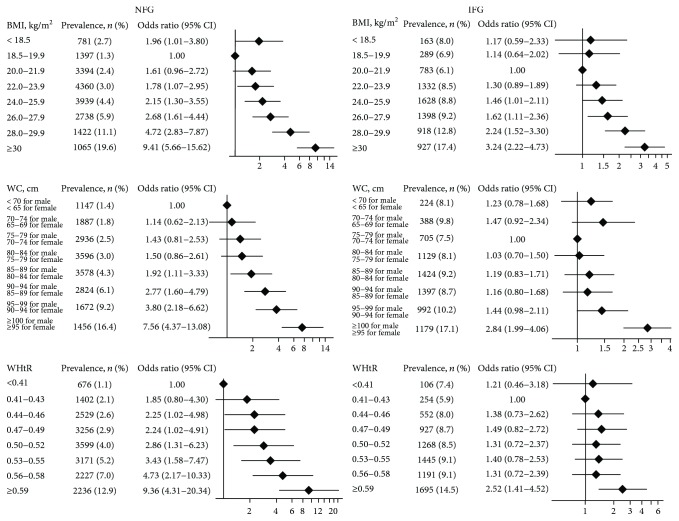
Logistic regression analyses of the effects of BMI, WC, and WHtR on insulin resistance. NFG: normal fasting glucose; IFG: impaired fasting glucose; BMI: body mass index; WC: waist circumference; WHtR: waist-to-height ratio. Model was adjusted for age, gender, education (less than high school, high school or equivalent, or college or above), marital status (married or unmarried), smoking status (current smoker, former smoker, or never smoked), alcohol use (currently, formerly, or never), physical activity (yes or no), TC, TG, LDL, HDL, family history of diabetes (yes or no), glucose, and HbA1c.

The prevalence of IR was the lowest in the NFG participants with BMI 18.5–19.9 (Figure 2, Table S2). A J-shaped association between BMI and IR showed among NFG participants (Figure 2, Table S3). As compared with participants whose BMI ranged from 18.5 to 19.9, those in the lowest BMI category (<18.5) had a significantly increased risk of IR (OR, 1.96; 95% CI, 1.01–3.80) and so did those in higher BMI categories. The lowest prevalence of IR was 6.1% in the IFG participants with a BMI from 20.0 to 21.9 (Figure 2, Table S2). We also observed a J-shaped association although it was not statistically significant. Compared to BMI from 20.0 to 21.9, a significant increase in the risk of IR was seen in higher BMI categories.

There was a significant direct linear relationship between WC and the risk of IR (*P* < 0.001 for linear trend) among the participants with NFG (Figure 2, Table S4). In the same, a direct linear trend was also observed between WHtR and the risk of IR (*P* < 0.001 for linear trend) in NFG (Figure 2, Table S5). For IFG participants, the relationship of WC and WHtR to the risk of IR did not present a regular change pattern. A significant increased risk of IR was observed only in the highest WC category (≥100 for male or ≥95 for female: OR, 2.84; 95% CI, 1.99–4.06) and in the highest WHtR category (≥0.59: OR, 2.52; 95% CI, 4.41–4.52) among the participants with IFG (Figure 2).

## 4. Discussion

In this nationally representative cross-sectional study, we described the level of insulin and HOMA-IR by different glycemic statuses, gender, and age. We explored the effects of BMI, WC, and WHtR on IR using multiple linear regression analysis and observed a J-shaped association between BMI and IR among NFG participants. Among NFG participants, the risk of IR increased with WC and WHtR, and the response was linear. However, the risk of IR did not present a regular change pattern for IFG participants.

Some studies have reported that the relationship between FPG and insulin levels presented an inverted U shape [25]. As FPG rises, the concentration of insulin levels increases in a progressive way. The progressive increase in various levels of insulin can be regarded as an adaptive response of the pancreas to offset the progressive deterioration in glucose homeostasis. When FPG exceeds a certain value, it is hard for the beta cell to maintain its rising rate of insulin secretion with a sharp decline of plasma insulin concentration [25]. Hence, compared with those with new-onset diabetes and IFG participants, previously diagnosed diabetes without any control measures showed higher levels of FPG and HbA1c but lower levels of insulin and HOMA-IR.

In the present study, the levels of insulin and HOMA-IR were higher in females than in males among participants with IFG and NFG, similar to the results from Tohidi et al. who studied 309 nonobese healthy Iranian subjects (124 males and 185 females) which found higher serum insulin levels in females [11]. However, on the contrary, an observational study (673 males and 849 females, Caucasian adults, all free of diabetes) found that age-adjusted fasting insulin levels were higher in males [13]. Another study including 2246 nondiabetic Spanish adults found that HOMA-IR levels were higher in males than in females [10]. Numerous genetic polymorphisms have been associated with pancreatic beta-cell function and related to peripheral IR. Some of these polymorphisms were strictly race-dependent, and others have a transethnic association [26]. Therefore, those differences may be due to the effects of race and ethnicity on glucose homeostasis and circulating insulin levels [12].

There was a decreased trend of insulin and HOMA-IR levels with aging for the participants with IFG and NFG. These findings were similar to several previous studies which included nondiabetes or nonobese healthy subjects [10, 11, 27]. However, a study including 1146 healthy males and females with normal glucose tolerance aging from 18 to 85 years showed that the relationship between insulin action and age has no statistical significance after adjusted for BMI. They concluded that age per se was not a significant cause of IR in healthy Europeans [14]. This phenomenon can be interpreted as follows. The relationship between insulin action and age is confounded by their own disease status such as obesity and diabetes. In addition, aging is often accompanied by changes in body composition, dietary habits, and physical activity, all of which can influence insulin sensitivity [14]. In our study, when adjusted for demographic characteristics, lifestyle factors, anthropological indexes, and clinical indexes, the negative association between insulin and HOMA-IR levels and age still existed in the participants with IFG and NFG. However, for previously diagnosed and newly diagnosed diabetes participants, the situation became complicated as the relationship between insulin and HOMA-IR levels and age is related to gender. Hence, more studies are needed to evaluate the relationship of insulin action to age and gender in different ethnic groups.

Evidence from large epidemiologic studies has shown the parallel escalation of obesity and diabetes [2, 3]. Both of these metabolic disorders are characterized by defects of insulin action [6]. It has received wide agreement that the potential cause of IR in obesity are the decreased storage capacity of adipocytes and the elevated plasma free fatty acid levels, which further lead to the accumulation of ectopic fat and lipotoxicity in livers and muscles [5, 28]. The Nurses' Health Study and the Health Professionals' Follow-Up Study found a significant positive association between BMI and risk of diabetes [29, 30]. In addition, weight gain was monotonically related to the risk of diabetes and the risk increased by 7.3% for every kilogram of weight gained [31]. Abdominal obesity has been shown to increase the risk of diabetes, and the increased risk can be largely explained by the association of IR with the accumulation of abdominal adipose tissue [32]. Abdominal fat can be characterized as either subcutaneous or visceral, and the amount of intra-abdominal or visceral fat best correlates with IR [32]. Studies have reported that WC may be a good reflection of the accumulation of abdominal subcutaneous or visceral fat [33]. Meta-analysis showed that WHtR was a good tool for adults to screen cardiometabolic risk factors in a population of various nationalities and ethnic groups [19]. Several cohort studies have shown WC and WHtR as good predictors of T2DM incidence [20, 34]. In the current study, we found that the risk of IR increased with WC and WHtR, and the response was linear among NFG participants. However, the risk of IR did not present a regular change pattern for IFG participants. This indicates that fasting blood glucose may be a confounding factor in the relationship of WC, WHtR to IR for IFG participants.

Interestingly, low body weight (BMI < 18.5) also showed a high risk for IR in the NFG participants. Low body weight primarily reflects the losses of adipose tissue and/or lean tissue. Adipose tissue plays a central role in regulating energy metabolic and glucose homeostasis through its secretion of various bioactive proteins [35]. Experiments have shown that subcutaneous adipose tissue secreted some bioactive factors that mediate improvement in the metabolic profile [36]. The Health Professionals Follow-Up Study showed that loss of hip girth was associated with an increased risk of diabetes [31]. A decrease in hip girth may primarily reflect a loss of lean tissue [37]. Studies have shown the wasting of leg muscle mass with an increased risk of diabetes [38]. Peripheral muscle wasting and/or low muscle mass may contribute to both diminish insulin clearance from muscle and low muscle lipoprotein lipase mass and activity with a concomitant reduction in the capacity of muscle to use fatty acids [39]. In addition, insulin plays an important role in the regulation of skeletal muscle protein turnover. It promotes protein deposition both by the inhibition of proteolysis and stimulation of protein synthesis [40]. Research work has demonstrated that a loss of lean mass due to altered amino acid utilization results in the subsequent release of nitrogenous metabolites that may impair insulin action [41]. In the present study, we observed a J-shaped association between BMI and IR and found that the lowest BMI category (<18.5) had a significantly elevated risk of IR among NFG participants. For IFG participants, we also observed a similar association although there was no statistical significance. Further analyses were needed to provide a better understanding for the relationship between BMI and IR among IFG participants.

There are also limitations in our study. The cross-sectional nature of the present study design means that the causality cannot be established between obesity and IR. However, the nationally representative data may indicate some relevance. More cohort studies are needed to evaluate the association between low body weight and IR in different glycemic statuses.

## 5. Conclusion

To our knowledge, the current study is the first one discussing the influence of different glycemic statuses on the relationship of insulin action to age, gender, BMI, WC, and WHtR among Chinese population. The levels of insulin and HOMA-IR were higher in females than in males among the participants with previously diagnosed diabetes, IFG, and NFG and decreased with age for IFG and NFG participants. A J-shaped association between BMI and IR was observed among NFG participants. Low body weight and overweight/obesity were risk factors for IR. The risk of IR increased with WC and WHtR, and the response was linear for the participants with NFG but not in those with IFG. Relationships of insulin action to age, gender, BMI, WC, and WHtR present diversely in different glycemic statuses among Chinese population.

## Figures and Tables

**Table 1:** Demographics and clinical characteristics of the participants by glycemic status.

	Overall (*n* = 35,327)	Previously diagnosed (*n* = 4360)	Newly diagnosed (*n* = 4433)	IFG (*n* = 7438)	NFG (*n* = 19,096)	*P* value
Age (years)	56.2 (56.1–56.4)	60.1 (59.8–60.4)^a,b,c^	57.0 (56.6–57.4)^a^	57.0 (56.7–57.3)^a^	54.8 (54.6–55.1)	<0.001
Gender, (male, %)	49.4 (49.0–49.8)	46.9 (46.7–47.1)^a,b,c^	51.1 (50.9–51.3)^a^	49.4 (49.1–49.6)	49.6 (49.4–49.8)	<0.001
BMI (kg/m^2^)	24.8 (24.8-24.8)	25.7 (25.6–25.8)^a^	25.6 (25.5–25.8)^a^	25.6 (25.5–25.7)^a^	24.1 (24.0-24.1)	<0.001
WC (cm)	84.8 (84.7–84.9)	88.2 (87.9–88.5)^a,b^	87.6 (87.3–88.0)^a,b^	86.9 (86.7–87.2)^a^	82.6 (82.4–82.7)	<0.001
WHtR	0.53 (0.53-0.53)	0.55 (0.54-0.55)^a^	0.54 (0.54-0.55)^a^	0.54 (0.54-0.54)^a^	0.52 (0.51-0.52)	<0.001
SBP (mmHg)	137.6 (137.4–137.8)	142.9 (142.3–143.6)^a,b^	141.9 (141.3–142.6)^a^	141.1 (140.6–141.6)^a^	134.0 (133.7–134.3)	<0.001
DBP (mmHg)	80.0 (79.9–80.1)	80.5 (80.2–80.8)^a,b,c^	82.2 (81.8–82.5)^a^	81.7 (81.4–82.0)^a^	78.7 (78.6–78.9)	<0.001
TC (mmol/L)	4.9 (4.9-4.9)	4.9 (4.9-5.0)^a,b,c^	5.1 (5.1-5.2)^a,b^	5.0 (5.0-5.1)^a^	4.8 (4.8-4.8)	<0.001
TG (mmol/L)	1.8 (1.7-1.8)	2.0 (2.0-2.0)^a,c^	2.3 (2.2-2.3)^a,b^	2.0 (1.9-2.0)^a^	1.5 (1.5-1.5)	<0.001
LDL (mmol/L)	3.1 (3.1-3.1)	3.1 (3.1-3.2)^a,b,c^	3.2 (3.2-3.3)^a^	3.2 (3.2-3.2)^a^	3.0 (3.0-3.0)	<0.001
HDL (mmol/L)	1.3 (1.2-1.3)	1.2 (1.2-1.2)^a,b,c^	1.2 (1.2-1.2)^a,b^	1.2 (1.2-1.3)^a^	1.3 (1.3-1.3)	<0.001
FPG (mmol/L)	6.3 (6.2-6.3)	8.4 (8.3-8.4)^a,b,c^	9.1 (9.0-9.1)^a,b^	6.4 (6.4-6.4)^a^	5.1 (5.1-5.1)	<0.001
HbA1c (%)	5.5 (5.4-5.5)	6.8 (6.7-6.8)^a,b^	6.7 (6.7-6.8)^a,b^	5.5 (5.5-5.5)^a^	4.9 (4.8-4.9)	<0.001
HbA1c (mmol/mol)	36.1 (35.9–36.2)	50.6 (50.0–51.2)	50.0 (49.4–50.7)	36.5 (36.4–36.7)	29.4 (29.3–29.5)	
Insulin	2.1 (2.0-2.1)	2.1 (2.1-2.2)^a,b,c^	2.5 (2.4-2.5)^a,b^	2.3 (2.3-2.3)^a^	1.9 (1.8-1.9)	<0.001
HOMA-IR	0.7 (0.7-0.8)	1.1 (1.1-1.1)^a,b,c^	1.5 (1.5-1.5)^a,b^	1.0 (1.0-1.0)^a^	0.4 (0.1–0.4)	<0.001

Mean values (95% confidence interval) or percentages (95% confidence interval) were shown. IFG: impaired fasting glucose; NFG: normal fasting glucose. Insulin and HOMA-IR values were logarithmically transformed. ^a^ *P* < 0.001 compared with NFG; ^b^ *P* < 0.001 compared with IFG; ^c^ *P* < 0.001 compared with newly diagnosed.

**Table 2:** Gender-specific and age-specific serum insulin levels and HOMA-IR in the participants by glycemic status.

	Overall		Previously diagnosed (*n* = 4360)		Newly diagnosed (*n* = 4433)		IFG (*n* = 7438)		NFG (*n* = 19,096)	
	Insulin	HOMA-IR	Insulin	HOMA-IR	Insulin	HOMA-IR	Insulin	HOMA-IR	Insulin	HOMA-IR
Gender										
Male	2.0 (2.0-2.0)	0.7 (0.6-0.7)	2.0 (2.0-2.1)	1.0 (0.9-1.0)	2.4 (2.4-2.5)	1.5 (1.4-1.5)	2.2 (2.2-2.2)	0.9 (0.9-1.0)	1.7 (1.7-1.8)	0.3 (0.2-0.3)
Female	2.2 (2.1-2.2)	0.8 (0.8-0.8)	2.2 (2.2-2.2)	1.2 (1.1-1.2)	2.5 (2.5-2.5)	1.5 (1.5-1.6)	2.4 (2.4-2.4)	1.1 (1.1-1.1)	2.0 (1.9-2.0)	0.5 (0.5-0.6)
*P* values †	<0.001	<0.001	<0.001	<0.001	0.101	0.012	<0.001	<0.001	<0.001	<0.001
Gender- and age- specific										
Male, age, y										
18–44	2.2 (2.2-2.3)	0.9 (0.8-0.9)	2.4 (2.3–2.5)	1.4 (1.2–1.5)	2.7 (2.6–2.8)	1.8 (1.7–1.9)	2.6 (2.6-2.7)	1.4 (1.3-1.4)	2.0 (2.0-2.0)	0.5 (0.5-0.5)
45–59	2.0 (2.0-2.0)	0.7 (0.7-0.7)	2.1 (2.0-2.1)	1.1 (1.0-1.1)	2.4 (2.4-2.5)	1.5 (1.4–1.6)	2.2 (2.2-2.2)	0.9 (0.9-1.0)	1.8 (1.7-1.8)	0.3 (0.2-0.3)
60–69	1.8 (1.8-1.9)	0.5 (0.5-0.5)	2.0 (1.9-2.0)	0.9 (0.8-0.9)	2.3 (2.2–2.4)	1.3 (1.3-1.4)	2.1 (2.0-2.1)	0.8 (0.8-0.8)	1.6 (1.6-1.6)	0.1 (0.1-0.1)
≥70	1.9 (1.9-2.0)	0.6 (0.5-0.6)	2.1 (2.0-2.1)	1.0 (0.9–1.1)	2.4 (2.3–2.5)	1.4 (1.3–1.5)	2.0 (2.0-2.1)	0.8 (0.7-0.8)	1.6 (1.6-1.7)	0.1 (0.1-0.1)
Model 1‡	<0.001	<0.001	0.028	0.001	<0.001	<0.001	<0.001	<0.001	<0.001	<0.001
Model 2‡	<0.001	<0.001	0.278	0.436	<0.001	<0.001	<0.001	<0.001	<0.001	<0.001
Model 3‡	<0.001	<0.001	0.725	0.977	<0.001	<0.001	<0.001	<0.001	<0.001	<0.001
Female, age, y										
18–44	2.3 (2.2-2.3)	0.9 (0.8-0.9)	2.3 (2.2–2.5)	1.2 (1.1–1.4)	2.6 (2.6-2.7)	1.7 (1.6–1.8)	2.7 (2.6-2.7)	1.4 (1.3–1.5)	2.1 (2.1-2.1)	0.6 (0.5-0.6)
45–59	2.1 (2.1-2.2)	0.8 (0.8-0.8)	2.2 (2.2-2.3)	1.2 (1.1-1.2)	2.4 (2.4-2.5)	1.5 (1.5-1.6)	2.3 (2.3-2.4)	1.1 (1.0-1.1)	2.0 (2.0-2.0)	0.5 (0.5-0.5)
60–69	2.1 (2.1-2.1)	0.8 (0.8-0.8)	2.2 (2.2-2.3)	1.1 (1.1-1.2)	2.5 (2.4-2.5)	1.5 (1.4–1.6)	2.3 (2.3-2.3)	1.0 (1.0-1.1)	1.9 (1.9-1.9)	0.4 (0.4-0.5)
≥70	2.1 (2.0-2.1)	0.8 (0.8-0.8)	2.3 (2.2-2.3)	1.2 (1.1–1.3)	2.5 (2.4-2.5)	1.5 (1.4–1.6)	2.3 (2.2-2.3)	1.0 (1.0-1.1)	1.8 (1.8-1.9)	0.4 (0.4-0.4)
Model 1‡	<0.001	<0.001	0.937	0.346	0.006	0.006	<0.001	<0.001	<0.001	<0.001
Model 2‡	<0.001	<0.001	0.959	0.822	0.011	0.014	<0.001	<0.001	<0.001	<0.001
Model 3‡	<0.001	<0.001	0.743	0.512	0.118	0.023	<0.001	<0.001	<0.001	<0.001

Mean values (95% confidence interval) were shown. IFG: impaired fasting glucose; NFG: normal fasting glucose. Insulin and HOMA-IR values were logarithmically transformed; ^†^ *P* values from ANCOVA; ^‡^ *P* values from multiple linear regression. Model 1 was adjusted for education (less than high school, high school or equivalent, or college or above), marital status (married or unmarried), smoking status (current smoker, former smoker, or never smoked), alcohol use (currently, formerly, or never), physical activity (yes or no), TC, TG, LDL, and HDL. Model 2 was adjusted for the variables in model 1 plus family history of diabetes (yes or no), glucose, and HbA1c. Model 3 was adjusted for the variables in model 2 plus BMI and WC.

## Data Availability

The data related to our study will be released in 2019 on the website of Chinese Center for Disease Control and Prevention.

## Supplementary Materials

Supplementary Materials Table S1: serum insulin and HOMA-IR levels in previously diagnosed diabetes except for insulin injection. Table S2: the ratio of insulin resistance by body mass index, waist circumference, and waist-to-height ratio in NFG and IFG. Table S3: logistic regression analysis of the effects of BMI on insulin resistance. Table S4: logistic regression analysis of the effects of WC on insulin resistance. Table S5: logistic regression analysis of the effects of WHtR on insulin resistance.
