# Supplementary material for: Relationships of Insulin Action to Age, Gender, Body Mass Index, and Waist Circumference Present Diversely in Different Glycemic Statuses among Chinese Population
Source: J Diabetes Res. 2018 Aug 23;2018:1682959. doi: 10.1155/2018/1682959 (PMC6126106; doi:10.1155/2018/1682959)
Supplement: Supplementary Materials — Table S1: serum insulin and HOMA-IR levels in previously diagnosed diabetes except for insulin injection. Table S2: the ratio of insulin resistance by body mass index, waist circumference, and waist-to-height ratio in NFG and IFG. Table S3: logistic regression analysis of the effects of BMI on insulin resistance. Table S4: logistic regression analysis of the effects of WC on insulin resistance. Table S5: logistic regression analysis of the effects of WHtR on insulin resistance. [file 1682959.f1.docx]

**Supplementary Materials**

**Table S1. Serum insulin and HOMA-IR levels in previously-diagnosed diabetes** **except for insulin injection.**

|  | Previously-diagnosed ^†^ (n=3378) | |
| --- | --- | --- |
|  | Insulin | HOMA-IR |
| Gender- and age- specific |  |  |
| Male, age, y |  |  |
| 18-44 | 2.4 (2.3-2.5) | 1.3 (1.2-1.5) |
| 45-59 | 2.0 (2.0-2.1) | 1.0 (0.9-1.0) |
| 60-69 | 1.8 (1.8-1.9) | 0.7 (0.6-0.8) |
| ≥70 | 1.9 (1.9-2.0) | 0.8 (0.7-0.9) |
| Model 1 | 0.001 | <0.001 |
| Model 2 | 0.024 | 0.051 |
| Model 3 | 0.134 | 0.237 |
| Female, age, y |  |  |
| 18-44 | 2.3 (2.1-2.4) | 1.1 (0.9-1.3) |
| 45-59 | 2.1 (2.1-2.2) | 1.1 (1.0-1.1) |
| 60-69 | 2.2 (2.1-2.2) | 1.1 (1.0-1.1) |
| ≥70 | 2.2 (2.1-2.2) | 1.1 (1.0-1.2) |
| Model 1 | 0.809 | 0.501 |
| Model 2 | 0.889 | 0.875 |
| Model 3 | 0.311 | 0.162 |

Mean values (95% confidence interval) are shown; Insulin and HOMA-IR values are logarithmically transformed; ^†^ except for insulin injection; *P* values from multiple linear regression. Model 1 was adjusted for education (less than high school, high school or equivalent, college or above), marital status (married or unmarried), smoking status (current smoker, former smoker, or never smoked), alcohol use (currently, formerly, or never), physical activity (yes or no), TC, TG, LDL and HDL. Model 2 was adjusted for the variables in model 1 plus family history of diabetes (yes or no), glucose, and HbA1c. Model 3 was adjusted for the variables in model 2 plus BMI and WC.

**Table S2.** **The ratio of Insulin Resistance by Body Mass Index, Waist Circumference and Waist-to-Height Ratio in NFG and IFG**

|  | NFG, n (%) | IFG, n (%) |
| --- | --- | --- |
| Body mass index, kg/m2 |  |  |
| <18.5 | 781 (2.7) | 163 (8.0) |
| 18.5-19.9 | 1397 (1.3) | 289 (6.9) |
| 20.0-21.9 | 3394 (2.4) | 783 (6.1) |
| 22.0-23.9 | 4360 (3.0) | 1332 (8.5) |
| 24.0-25.9 | 3939 (4.4) | 1628 (8.8) |
| 26.0-27.9 | 2738 (5.9) | 1398 (9.2) |
| 28.0-29.9 | 1422 (11.1) | 918 (12.8) |
| ≥30 | 1065 (19.6) | 927 (17.4) |
| Waist circumference |  |  |
| <70 for male (<65 for female) | 1147 (1.4) | 224 (8.1) |
| 70-74 for male (65-69 for female) | 1887 (1.8) | 388 (9.8) |
| 75-79 for male (70-74 for female) | 2936 (2.5) | 705 (7.5) |
| 80-84 for male (75-79 for female) | 3596 (3.0) | 1129 (8.1) |
| 85-89 for male (80-84 for female) | 3578 (4.3) | 1424 (9.2) |
| 90-94 for male (85-89 for female) | 2824 (6.1) | 1397 (8.7) |
| 95-99 for male (90-94 for female) | 1672 (9.2) | 992 (10.2) |
| ≥100 for male (≥95 for female) | 1456 (16.4) | 1179 (17.1) |
| Waist-to-Height Ratio |  |  |
| <0.41 | 676 (1.1) | 106 (9.4) |
| 0.41-0.43 | 1402 (2.1) | 254 (5.9) |
| 0.44-0.46 | 2529 (2.6) | 552 (8.0) |
| 0.47-0.49 | 3256 (2.9) | 927 (8.7) |
| 0.50-0.52 | 3599 (4.0) | 1268 (8.5) |
| 0.53-0.55 | 3171 (5.2) | 1445 (9.1) |
| 0.56-0.58 | 2227 (7.0) | 1191 (9.1) |
| ≥0.59 | 2236 (12.9) | 1695 (14.5) |

IFG, impaired fasting glucose; NFG, normal fasting glucose; IR was defined using HOMA-IR >95th percentile for NFG and HOMA-IR >90th percentile for IFG.

**Table S3. Logistic regression analysis of the effects of BMI on insulin resistance**

|  | Model 1 | Model 2 | Model 3 |
| --- | --- | --- | --- |
| NFG |  |  |  |
| <18.5 | 2.18 (1.15-4.12) | 2.02 (1.06-3.86) | 1.96 (1.01-3.80) |
| 18.5-19.9 | 1.00 | 1.00 | 1.00 |
| 20.0-21.9 | 1.94 (1.16-3.26) | 1.75 (1.04-2.93) | 1.61 (0.96-2.72) |
| 22.0-23.9 | 2.50 (1.52-4.11) | 1.97 (1.19-3.25) | 1.78 (1.07-2.95) |
| 24.0-25.9 | 3.78 (2.32-6.17) | 2.53 (1.54-4.17) | 2.15 (1.30-3.55) |
| 26.0-27.9 | 5.06 (3.09-8.28) | 3.15 (1.91-5.21) | 2.68 (1.61-4.44) |
| 28.0-29.9 | 9.94 (6.06-16.29) | 5.89 (3.55-9.77) | 4.72 (2.83-7.87) |
| ≥30 | 19.23 (11.78-31.40) | 11.41 (6.91-18.83) | 9.41 (5.66-15.62) |
| IFG |  |  |  |
| <18.5 | 1.36 (0.71-2.60) | 1.26 (0.65-2.43) | 1.17 (0.59-2.33) |
| 18.5-19.9 | 1.16 (0.67-2.01) | 1.08 (0.62-1.88) | 1.14 (0.64-2.02) |
| 20.0-21.9 | 1.00 | 1.00 | 1.00 |
| 22.0-23.9 | 1.41 (0.99-2.01) | 1.34 (0.93-1.91) | 1.30 (0.89-1.89) |
| 24.0-25.9 | 1.46 (1.04-2.05) | 1.34 (0.94-1.90) | 1.46 (1.01-2.11) |
| 26.0-27.9 | 1.56 (1.10-2.20) | 1.38 (0.96-1.98) | 1.62 (1.11-2.36) |
| 28.0-29.9 | 2.16 (1.51-3.08) | 1.85 (1.28-2.68) | 2.24 (1.52-3.30) |
| ≥30 | 2.92 (2.08-4.11) | 2.48 (1.73-3.55) | 3.24 (2.22-4.73) |

IFG, impaired fasting glucose; NFG, normal fasting glucose; IR was defined using HOMA-IR >95th percentile for NFG and HOMA-IR >90th percentile for IFG; Model 1 was adjusted for age gender. Model 2 was adjusted for the variables in model 1 plus education (less than high school, high school or equivalent, college or above), marital status (married or unmarried), smoking status (current smoker, former smoker, or never smoked), alcohol use (currently, formerly, or never), physical activity (yes or no), TC, TG, LDL and HDL. Model 3 was adjusted for the variables in model 2 plus family history of diabetes (yes or no), glucose, and HbA1c.

**Table S4. Logistic regression analysis of the effects of WC on insulin resistance**

|  | Model 1 | Model 2 | Model 3 |
| --- | --- | --- | --- |
| NFG |  |  |  |
| <70 for male (<65 for female) | 1.00 | 1.00 | 1.00 |
| 70-74 for male (65-69 for female) | 1.31 (0.71-2.42) | 1.21 (0.65-2.24) | 1.14 (0.62-2.13) |
| 75-79 for male (70-74 for female) | 1.89 (1.08-3.31) | 1.59 (0.91-2.80) | 1.43 (0.81-2.53) |
| 80-84 for male (75-79 for female) | 2.32 (1.34-4.01) | 1.71 (0.98-2.97) | 1.50 (0.86-2.61) |
| 85-89 for male (80-84 for female) | 3.50 (2.05-5.98) | 2.33 (1.35-4.01) | 1.92 (1.11-3.33) |
| 90-94 for male (85-89 for female) | 5.11 (3.00-8.72) | 3.23 (1.87-5.57) | 2.77 (1.60-4.79) |
| 95-99 for male (90-94 for female) | 8.02 (4.68-13.73) | 4.63 (2.67-8.02) | 3.80 (2.18-6.62) |
| ≥100 for male (≥95 for female) | 16.03 (9.43-27.27) | 9.11 (5.29-15.68) | 7.56 (4.37-13.08) |
| IFG |  |  |  |
| <70 for male (<65 for female) | 1.25 (0.79-1.70) | 1.24 (0.78-1.67) | 1.23 (0.78-1.68) |
| 70-74 for male (65-69 for female) | 1.45 (0.93-2.26) | 1.48 (0.94-2.33) | 1.47 (0.92-2.34) |
| 75-79 for male (70-74 for female) | 1.00 | 1.00 | 1.00 |
| 80-84 for male (75-79 for female) | 1.09 (0.76-1.56) | 1.04 (0.72-1.49) | 1.03 (0.70-1.50) |
| 85-89 for male (80-84 for female) | 1.30 (0.93-1.82) | 1.15 (0.82-1.63) | 1.19 (0.83-1.71) |
| 90-94 for male (85-89 for female) | 1.25 (0.89-1.76) | 1.10 (0.77-1.56) | 1.16 (0.80-1.68) |
| 95-99 for male (90-94 for female) | 1.52 (1.06-2.16) | 1.32 (0.91-1.89) | 1.44 (0.98-2.11) |
| ≥100 for male (≥95 for female) | 2.70 (1.96-3.74) | 2.26 (1.61-3.18) | 2.84 (1.99-4.06) |

IFG, impaired fasting glucose; NFG, normal fasting glucose; IR was defined using HOMA-IR >95th percentile for NFG and HOMA-IR >90th percentile for IFG; Model 1 was adjusted for age gender. Model 2 was adjusted for the variables in model 1 plus education (less than high school, high school or equivalent, college or above), marital status (married or unmarried), smoking status (current smoker, former smoker, or never smoked), alcohol use (currently, formerly, or never), physical activity (yes or no), TC, TG, LDL and HDL. Model 3 was adjusted for the variables in model 2 plus family history of diabetes (yes or no), glucose, and HbA1c.

**Table S5. Logistic regression analysis of the effects of WHtR on insulin resistance**

|  | Model 1 | Model 2 | Model 3 |
| --- | --- | --- | --- |
| NFG |  |  |  |
| <0.41 | 1.00 | 1.00 | 1.00 |
| 0.41-0.43 | 2.09 (0.91-4.82) | 2.01 (0.87-4.64) | 1.85 (0.80-4.30) |
| 0.44-0.46 | 2.92 (1.33-6.42) | 2.54 (1.15-5.59) | 2.25 (1.02-4.98) |
| 0.47-0.49 | 3.40 (1.56-7.38) | 2.65 (1.21-5.78) | 2.24 (1.02-4.91) |
| 0.50-0.52 | 4.96 (2.31-10.69) | 3.51 (1.62-7.60) | 2.86 (1.31-6.23) |
| 0.53-0.55 | 6.81 (3.17-14.64) | 4.38 (2.02-9.50) | 3.43 (1.58-7.47) |
| 0.56-0.58 | 9.79 (4.54-21.08) | 6.01 (2.77-13.06) | 4.73 (2.17-10.33) |
| ≥0.59 | 20.78 (9.70-44.50) | 12.14 (5.62-26.25) | 9.36 (4.31-20.34) |
| IFG |  |  |  |
| <0.41 | 1.18 (1.46-3.04) | 1.05 (0.40-2.74) | 1.21 (0.46-3.18) |
| 0.41-0.43 | 1.00 | 1.00 | 1.00 |
| 0.44-0.46 | 1.44 (0.78-2.67) | 1.39 (0.75-2.59) | 1.38 (0.73-2.62) |
| 0.47-0.49 | 1.70 (0.96-3.04) | 1.62 (0.90-2.90) | 1.49 (0.82-2.72) |
| 0.50-0.52 | 1.65 (0.94-2.91) | 1.44 (0.80-2.56) | 1.31 (0.72-2.37) |
| 0.53-0.55 | 1.86 (1.06-3.26) | 1.59 (0.90-2.83) | 1.40 (0.78-2.53) |
| 0.56-0.58 | 1.91 (1.08-3.37) | 1.56 (0.87-2.79) | 1.31 (0.72-2.39) |
| ≥0.59 | 3.45 (1.99-5.97) | 2.82 (1.60-4.98) | 2.52 (1.41-4.52) |

IFG, impaired fasting glucose; NFG, normal fasting glucose; IR was defined using HOMA-IR >95th percentile for NFG and HOMA-IR >90th percentile for IFG; Model 1 was adjusted for age gender. Model 2 was adjusted for the variables in model 1 plus education (less than high school, high school or equivalent, college or above), marital status (married or unmarried), smoking status (current smoker, former smoker, or never smoked), alcohol use (currently, formerly, or never), physical activity (yes or no), TC, TG, LDL and HDL. Model 3 was adjusted for the variables in model 2 plus family history of diabetes (yes or no), glucose and HbA1c.
